# Maternal Exposure to 2,4-Di-tert-butylphenol During Pregnancy in a Mouse Model Leads to Abnormal Development of the Urinary System in Offspring

**DOI:** 10.3390/toxics13110991

**Published:** 2025-11-18

**Authors:** Yuanyan Jiang, Ningli Ye, Minghui Yu, Haixin Ju, Chunyan Wang, Hengmin Wang, Jiaojiao Liu, Qian Shen, Hong Xu

**Affiliations:** 1 Department of Nephrology, Children’s Hospital of Fudan University, National Children’s Medical Center, Shanghai 201102, China; 22111240013@m.fudan.edu.cn (Y.J.); 23211240022@m.fudan.edu.cn (N.Y.); 16211240027@fudan.edu.cn (M.Y.); 24111240014@m.fudan.edu.cn (H.J.); 22111240037@m.fudan.edu.cn (C.W.); 23111240041@m.fudan.edu.cn (H.W.); 122111240030@fudan.edu.cn (J.L.); 2 Shanghai Kidney Development & Pediatric Kidney Disease Research Center, Shanghai 201102, China; 3 National Key Laboratory of Kidney Diseases, Beijing 100853, China

**Keywords:** 2,4-DTBP, endocrine-disrupting chemicals, maternal exposure, maternal–fetal transfer, CAKUT, vesicoureteral reflux, hydronephrosis, litter effect, RXRα

## Abstract

The occurrence of congenital anomalies of the kidney and urinary tract (CAKUT) is influenced by intrauterine environmental factors, and maternal exposure to endocrine-disrupting chemicals (EDCs) during pregnancy may affect the kidney development of offspring. 2,4-Di-tert-butylphenol (2,4-DTBP) is a high-production volume chemical classified as an EDC, which has been detected in humans and has been found to increase mortality and malformation rates in zebrafish embryos. Its effects on mammalian development are still unknown. In this study, a maternal mouse model exposed to 2,4-DTBP throughout pregnancy was established by gavage. The overall conditions of the maternal mice and their offspring were observed, and the concentrations of 2,4-DTBP in maternal serum and offspring tissues were measured using liquid chromatography–tandem mass spectrometry. Exposure to 2,4-DTBP of 75 µg/g·day during pregnancy markedly reduced the early pregnancy rate in mice to 41.75% (95% CI: 33.53–49.97%; *n* = 139), compared to 82.29% (95% CI: 74.18–90.39%; *n* = 85) in the controls (*p* < 0.0001), with a relative risk (RR) of 0.51 (95% CI: 0.41–0.63). 2,4-DTBP could accumulate in maternal mice and be transferred to embryos and internal organs of the offspring, and is associated with the elevated risk of CAKUT in the offspring, primarily manifesting as hydronephrosis/ureteral dilation. The CAKUT rate of DTBP-75 group is 33.59% (95% CI: 17.62–49.56%; N = 9, *n* = 56), compared to 11.85% (95% CI: 2.43–21.28%; N = 9, *n* = 67) in the controls (*p* = 0.02), RR = 2.53 (95% CI: 1.18–5.42). These findings enhance the understanding of the health risks posed by 2,4-DTBP and provide a theoretical basis for environmental monitoring in public health.

## 1. Introduction

Congenital anomalies of the kidney and urinary tract (CAKUT) are common birth defects, accounting for 20–30% of prenatal developmental anomalies [1]. Single-gene mutations can explain only 6–20% of cases, with intrauterine environmental factors also playing a significant role [2]. Previous studies have already shown that maternal health status [3], medication use [4], and nutrition [5] during pregnancy significantly influence offspring kidney development and the risk of CAKUT. Studies demonstrated that maternal occupational exposure to alkylphenolic compounds, phthalates, and other endocrine-disrupting chemicals (EDCs) during pregnancy might have potential associations with CAKUT [6,7].

2,4-Di-tert-butylphenol (2,4-DTBP), an alkylphenol compound and emerging EDC, is widely used as a synthetic phenolic antioxidant (SPA) in plastics, automotive components, food packaging, and personal care products [8]. 2,4-DTBP is already classified as a high-production volume chemical [9]. It leaks into aquatic ecosystems and accumulates in aquatic organisms [10]. In addition to the bioaccumulation effects, human exposure also occurs via indoor dust, personal care products, and food packaging migration [11]. 2,4-DTBP has been detected in a range of human biological fluids, including serum samples [12], urine [13], and breast milk [14]. It shows universal presence in maternal blood, placenta, and umbilical cord blood with moderate maternal–fetal transfer capacity [15]. Consequently, maternal exposure to 2,4-DTBP during pregnancy poses a risk of intrauterine exposure for offspring as well. However, current research has not yet established whether intrauterine exposure to 2,4-DTBP leads to increased concentrations in offspring.

Given its widespread environmental presence and high detection frequency, 2,4-DTBP has raised significant concerns regarding its potential toxicity. In aquatic plants, studies indicate that 2,4-DTBP disrupts chloroplast ultrastructure, impairs metabolic activities, and reduces photosynthetic performance [16,17]. At environmentally relevant concentrations, 2,4-DTBP can accumulate in the gills and digestive glands of the benthic aquatic organism Asian clam (*Corbicula fluminea*), leading to increased damage in the digestive tract and inducing cellular DNA damage [18]. Zebrafish embryos exposed to 2,4-DTBP exhibit developmental toxicity even at low concentrations, including elevated mortality, delayed hatching, and malformations such as spinal curvature and yolk sac edema. And the toxic effect on zebrafish embryos becomes more pronounced with elevated doses [19]. Research gaps persist on its developmental impacts in mammalian embryos, particularly kidney-related effects. Thus, this study investigated maternal serum accumulation, maternal–fetal transfer of 2,4-DTBP, and phenotypic outcomes in offspring following gestational exposure, to evaluate whether such exposure is associated with elevated neonatal renal tissue concentrations and an increased probability of CAKUT.

According to European Chemical Agency, in rats, the No-Observed-Adverse-Effect Level (NOAEL) for subchronic oral exposure to 2,4-DTBP was determined to be 150 µg/g·day [20,21]. Considering the species-specific dose conversion (approximately 1.5:1 for mouse-to-rat) and the relative vulnerability of pregnancy, pregnant mice received subchronic gavage doses of 75 or 15 µg/g·day throughout gestation, which correspond to 1/3 and 1/15 of the NOAEL, respectively. These two doses correspond to human daily intakes of 6 mg/kg and 1.2 mg/kg of 2,4-DTBP, respectively. Despite its exceeding over environmental levels (up to 0.3 mg/L in surface water [22]), evidence of its dose-dependent toxicity in zebrafish embryos [19], which underscores the significance of our study in highlighting the potential hazards posed by 2,4-DTBP.

## 2. Materials and Methods

### 2.1. Chemicals

2,4-DTBP (purity: 99%) and dimethyl sulfoxide (DMSO) were obtained from Sigma-Aldrich (Shanghai, China). Polyethylene glycol 300 (PEG 300) and polysorbate 80 (Tween 80) were obtained from Selleck Chemicals (Shanghai, China).

### 2.2. Animals and Experimental Schemes

This study utilized Hoxb7/myr-Venus mice (a generous gift from Prof. Costantini) on the FVB/NJ background, with green fluorescence labeling in the urothelium. All animals were housed in an SPF-grade facility, and the experimental protocol was approved by the Animal Care and Use Committee of Institute of Developmental Biology and Molecular Medicine (Protocol ID: IDM2024099). All procedures were conducted in compliance with the guidelines of the committee.

**Figure 1:**
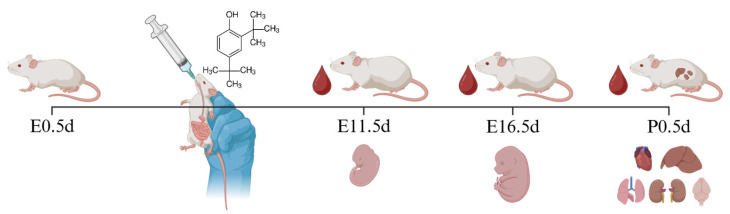
Schematic diagram of mouse model construction (Created in BioRender. Jiang, Y. (2025) https://app.biorender.com/illustrations/6727a44ca69bb5920a8b4d9c?slideId=b4aec76f-227d-4955-98d2-8b839eeda186).

**Figure 2:**
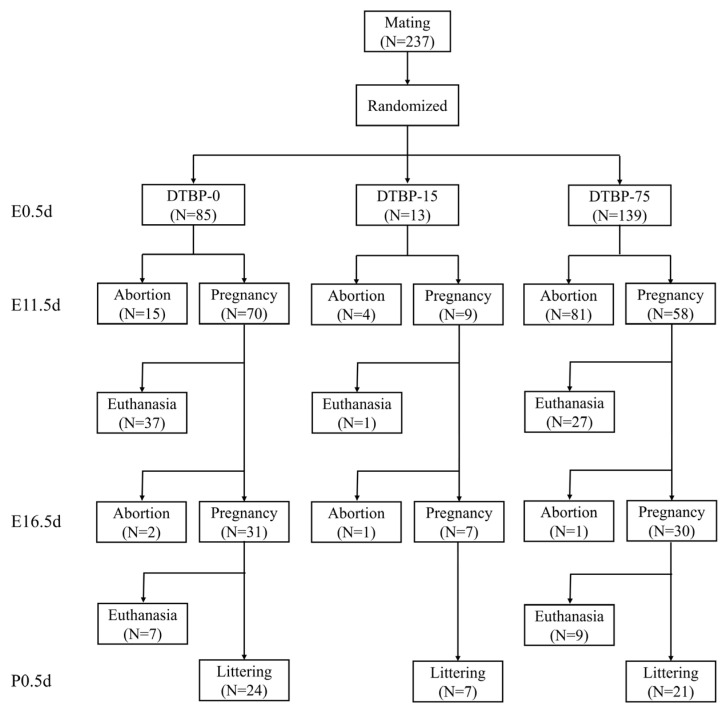
Flow diagram of the study.

Sexually mature female mice (8–12 weeks old) were used in the present study. Mice were paired at a female-to-male ratio of 2:1 in the evening, and the day a vaginal plug was observed was designated as embryonic 0.5 day (E0.5d). Female mice were randomly assigned to different groups, and received daily oral gavage for 19 consecutive days throughout the entire pregnancy (E0.5d–E18.5d) (Figure 1). All groups received a daily intragastric gavage of the same vehicle solution at a volume of 5 µL/g·day. The vehicle was composed of ultrapure water containing 1% DMSO, 40% PEG 300, and 5% Tween 80. For the treatment groups (DTBP-75 and DTBP-15), the vehicle was supplemented with 75 or 15 µg/g·day of 2,4-DTBP, respectively, while the control group (DTBP-0) received the vehicle without supplementation. The dosage was determined based on the NOAEL of 2,4-DTBP [20,21]. All solutions were freshly prepared each day. During the experiment, a subset of mice in DTBP-75 and DTBP-15 group was randomly selected at E11.5d, E16.5d, and postnatal 0.5 day (P0.5d) for blood collection and euthanasia (Figure 2). Pregnancy was confirmed based on a sustained increase in body weight or direct dissection, respectively.

### 2.3. Measurement of Liver and Kidney Function

Blood samples were collected from the mandibular vein of the maternal mice and centrifuged at 2500 rpm for 5 min. Serum was obtained and measured using a fully automated biochemical analyzer (Rayto, Shenzhen, China).

### 2.4. Sample Collection

Blood samples were collected from the maternal mice at E11.5d, E16.5d, and P0.5d, followed by cervical dislocation for euthanasia. The embryos were collected. P0.5d neonatal mice were euthanized by cervical dislocation, and the organs were collected. All samples were immediately frozen and stored at −80 °C.

### 2.5. Sample Preparation

For serum samples, 50 µL serum was transferred into a 2 mL chromatography vial, followed by the addition of 1 mL of n-hexane. The mixture was shaken at 300 rpm for 20 min, then subjected to ultrasonic treatment in an ice bath and centrifuged at 8000× *g* for 15 min. The supernatant was extracted again by adding 1 mL of n-hexane to the precipitate and this process was repeated twice. The supernatants were combined and were concentrated under nitrogen. The residue was re-dissolved in 150 µL of methanol and centrifuged at 8000× *g* for 30 min. Finally, 100 µL of the supernatant was transferred to a chromatography vial containing a glass liner for analysis.

For tissue samples, 500 µL 80% methanol solution pre-cooled at −20 °C was added as the extraction solution. The tissue was homogenized, and the homogenate was transferred to a 2 mL chromatography vial, and all subsequent procedures were the same as for the serum samples.

### 2.6. Liquid Chromatography–Tandem Mass Spectrometry (LC-MS) Analysis

Quantitative analysis of 2,4-DTBP was performed using LC-MS. The detection method was adapted from Du et al. [20]. Chromatographic separation was performed using an XBridge BEH C18 analytical column (Waters, Shanghai, China), with a trapping column (Waters, Shanghai, China) used for background reduction. The detailed LC parameters and gradient conditions are outlined in Table 1 and Table 2. The mass spectrometer (Shanghai, China) was operated in negative multiple reaction monitoring (MRM) mode, with detailed parameters provided in Table 3 and representative chromatograms in Supplementary Materials (Figure S1). A standard curve was generated using the external standard method for quantification, with a correlation coefficient (R^2^) of 0.9995. Tissue data were normalized by wet weight (ng/g), while serum data were normalized by volume (ng/L). Representative LC-MS chromatograms are provided in the Supplementary Materials (Figure S2).

### 2.7. Hematoxylin-Eosin (H&E) Staining

The tissues were fixed in 4% paraformaldehyde. After dehydration through a gradient series of ethanol and xylene, the tissues were embedded in paraffin, sectioned into thin slices, and dewaxed. The sections were rehydrated with decreasing concentrations of ethanol, stained with hematoxylin and eosin, and then dehydrated and sealed. The structural changes in the tissues were observed under a light microscope and assessed in a blinded manner.

### 2.8. Alcian Blue and Alizarin Red Staining

Embryos at E18.5d were fixed in 95% ethanol, treated with acetone, and stained with alcian blue and alizarin red solution. After treatment with 1% KOH, the embryos were treated in 20% glycerol for 1 week, and transferred to 50% glycerol.

### 2.9. CAKUT Assessment and Vesicoureteral Reflux (VUR) Test

A midline abdominal incision was made on P0.5d neonatal mice after euthanasia by cervical dislocation. The entire urinary system, including the kidneys, ureters, and bladder, was carefully dissected and photographed. Phenotypic assessment of these images was performed in a blinded manner by two independent investigators. Hydronephrosis was defined by the presence of increased renal pelvic translucency accompanied by ureteral tortuosity. Ureteral dilation was identified by significant ureteral tortuosity with loss of normal morphology or by dilation of the ureter at the renal pelvic junction (≥300 µm), in the absence of increased pelvic translucency. Both hydronephrosis and ureteral dilation were considered anomalies of the collecting system. A duplex kidney was diagnosed based on an abnormal indentation of the renal cortex, with or without the presence of a duplicated ureter. For VUR test, 20–25 µL of methylene blue dye (1 mg/mL) was manually injected slowly (over 3–5 s) into the bladder using an insulin syringe until the dye was expelled through the urethra or until visible bladder filling was achieved [23]. The retrograde flow of the dye into the kidneys via the ureters was monitored under a microscope. Images were acquired 5 min post-injection and evaluated by investigators blinded to the experimental groups. VUR was defined as positive upon clear observation of dye within the renal pelvis.

### 2.10. Statistical Analysis

Data measured by LC-MS were processed using Sciex OS 1.4 software. For continuous variables, statistical analysis was performed using a two-tailed unpaired *t* test. For binary variables, statistical analysis was performed using Fisher’s exact test. These statistical analysis were processed using GraphPad Prism 10.0. When calculating the early pregnancy rate, the generalized estimating equations (GEE) model with a log link (Poisson regression with robust variance) was used and the maternal body weight was included as a covariate. In addition, the data was analyzed with litter treated as a random effect to account for intra-litter correlation. For the analysis of crown-rump length of the embryos, and the newborn body weight, a linear mixed-effects model (LMM) was applied. The GEE model with a log link, and robust standard errors (SE) clustered by litter to analyze the CAKUT rate. The sex-stratified results, and multiple-comparison correction across phenotypes (hydronephrosis, ureteral dilation, and duplex kidney) were applied using Bonferroni, Holm, and FDR methods. The adjusted *p*-values were reported accordingly. The results were calculated in STATA 18. The results were presented as the mean ± SE or rate with 95% confidence interval (CI) alongside *p*-values. The relative risk (RR) was also reported to quantify differences. A *p*-value ≥ 0.05 was considered non-significant (ns), and a *p*-value < 0.05 was considered statistically significant.

A priori power analysis was conducted to determine the minimum number of litters required per group to detect a meaningful difference in the incidence of CAKUT. Based on preliminary published literature [24], CAKUT incidence in the DTBP-0 group was assumed to be 15%. With a significance level of 0.05, a desired statistical power of 80%, a relative risk of 3 and the average number of pups per litter of 7, calculations indicated that at least 9 litters per group would be needed.

## 3. Results

### 3.1. Exposure to 2,4-DTBP During Pregnancy Is Associated with the Risk of Early Pregnancy Loss (EPL)

**Figure 3:**
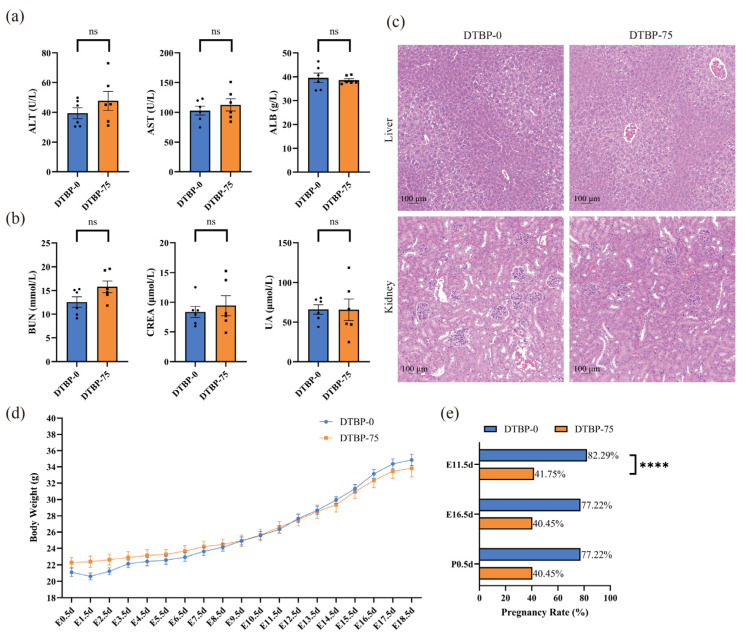
The effect of the whole pregnancy exposure to 2,4-DTBP. Liver (**a**) and kidney function (**b**) in maternal mice at 0.5 day postpartum. Liver function was assessed by measuring alanine transaminase (ALT), aspartate aminotransferase (AST), and albumin (ALB), while kidney function was evaluated by analyzing blood urea nitrogen (BUN), serum creatinine (CREA), and uric acid (UA) (*n* = 6). (**c**) H&E stained hepatic and renal tissue sections from 0.5 day postpartum maternal mice. (**d**) Maternal body weight during gestation in mice (*n* = 9). (**e**) Pregnancy rate in maternal mice adjusted by maternal body weight (per female). ns, non-significant. **** *p* < 0.0001 indicates a significant difference.

No differences in liver or kidney function were observed between the DTBP-75 group and the DTBP-0 group of maternal mice at P0.5d (*n* = 6; Figure 3a,b). Histological abnormalities in the kidney and liver were not observed (Figure 3c). Throughout pregnancy, no significant differences in maternal body weight were observed among the dams that successfully delivered offspring (Figure 3d). However, exposure to 2,4-DTBP at 75 µg/g·day significantly compromised early pregnancy maintenance. Pregnancy status was assessed at early pregnancy (E11.5d), mid-pregnancy (E16.5d), and postnatal (P0.5d) stages. After adjustment for maternal body weight as a covariate using GEE model, the DTBP-75 group showed a markedly reduced early pregnancy rate (41.75%; 95% CI: 33.53–49.97%; *n* = 139) versus the DTBP-0 controls (82.29%; 95% CI: 74.18–90.39%; *n* = 85; *p* < 0.0001), RR = 0.51 (95% CI: 0.41–0.63) (Figure 3e). No further decline in the pregnancy rate was observed during mid-pregnancy or beyond.

### 3.2. The Effects of Intrauterine Exposure to 2,4-DTBP on the Number and Size of Offspring

**Figure 4:**
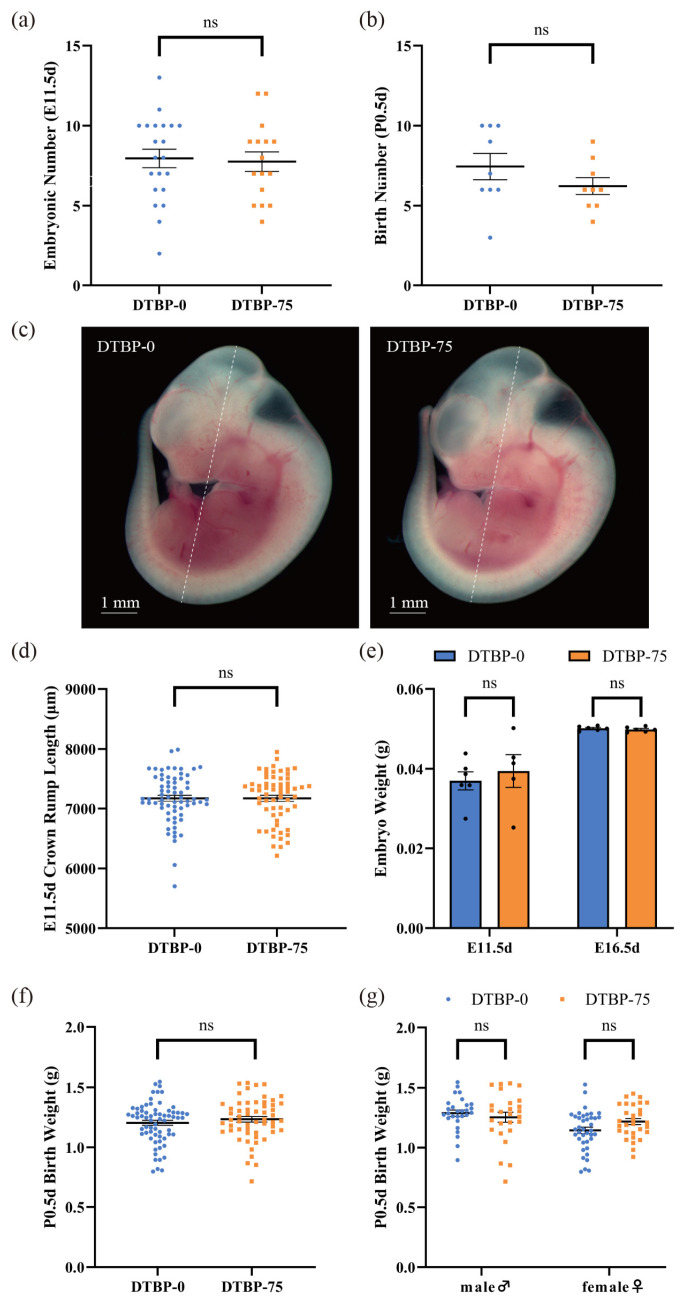
Effects of intrauterine exposure to 2, 4-DTBP on litter size and growth parameters. Embryonic number at E11.5d (**a**) (N = 21, 16, respectively) and litter size at P0.5d (**b**) (N = 9). (**c**) The representative images of E11.5d embryos, the white dotted line indicates the crown-rump length. (**d**) The crown-rump length of E11.5d embryos (*n* = 68 from 8 litters, 62 from 9 litters, respectively). (**e**) The embryo weight at E11.5d (*n* = 6, 5, respectively) and E16.5d (*n* = 6). (**f**) The body weight at P0.5d (*n* = 67 from 9 litters, 56 from 9 litters, respectively). (**g**) The P0.5d body weight of male (*n* = 28, 26, respectively) and female mice (*n* = 39, 30, respectively). ns, non-significant.

No differences were observed in the number of embryos at E11.5d between the DTBP-0 group and the DTBP-75 group (7.95 ± 0.58 vs. 7.75 ± 0.61, *p* = 0.81; N = 21, 16, respectively; Figure 4a), and no notable changes in the number of offspring were observed at P0.5d (7.44 ± 0.82 vs. 6.22 ± 0.52, *p* = 0.23; N = 9; Figure 4b). The crown-rump length of the embryos at E11.5d was not significantly different (7174.42 ± 50.38 vs. 7174.55 ± 52.45 µm, *p* = 1.00; *n* = 68 from 8 litters, 62 from 9 litters, respectively; Figure 4c,d). A linear mixed-effects model, incorporating litter as a random effect, was used to account for litter structure. This analysis also revealed no significant difference in the adjusted crown-rump length at E11.5d between the DTBP-0 (7190.07 ± 85.51 µm) and DTBP-75 (7157.90 ± 83.24 µm) groups (*p* = 0.79). No notable changes in embryo weight were noted at E11.5d or E16.5d (0.0370 ± 0.0023 vs. 0.0394 ± 0.0041 g, *p* = 0.60; *n* = 6, 5, respectively; 0.0501 ± 0.0002 vs. 0.0498 ± 0.0003, *p* = 0.40; *n* = 6; Figure 4e). At P0.5d, the body weight of the newborn mice had no significant difference between two groups (1.2036 ± 0.0208 vs. 1.2340 ± 0.0234 g, *p* = 0.33; *n* = 67 from 9 litters, 56 from 9 litters, respectively; Figure 4f) and was not influenced by sex (Figure 4g). The litter-adjusted body weights of neonatal mice were 1.2269 ± 0.0477 and 1.2317 ± 0.0479 g, respectively (*p* = 0.94), also with no distinct differences.

### 3.3. Bioaccumulation and Maternal–Fetal Transfer of 2,4-DTBP

**Figure 5:**
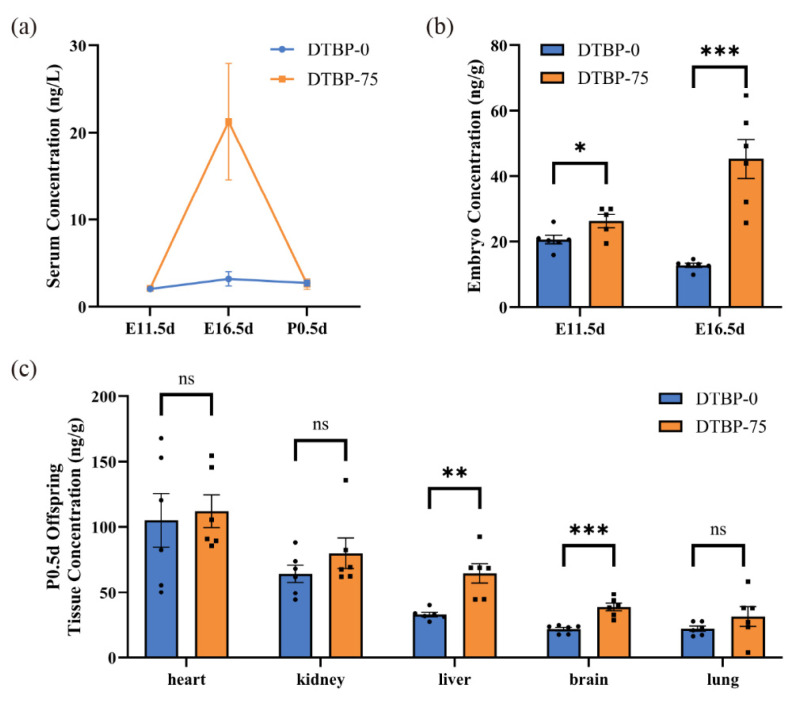
Concentration of 2,4-DTBP detected by LC-MS. (**a**) Serum 2,4-DTBP concentrations of pregnant mice at E11.5d (*n* = 5), E16.5d (*n* = 6) and P0.5d (*n* = 6). (**b**) Embryo 2,4-DTBP concentrations at E11.5d (*n* = 6, 5, respectively) and E16.5d (*n* = 6). (**c**) Tissue 2,4-DTBP concentrations of offspring at P0.5d (*n* = 6). ns, non-significant. *** *p* < 0.001, ** *p* < 0.01 and * *p* < 0.05 indicate significant differences.

Quantitative analysis of 2,4-DTBP was performed on the serum of maternal mice at E11.5d, E16.5d and P0.5d (Figure 5a). During mid-pregnancy (E16.5d), the serum 2,4-DTBP level in the DTBP-75 group reached 21.2385 ± 6.6883 ng/L (*n* = 6), which was significantly greater than that in early pregnancy (E11.5d), with a 6.6-fold increase compared with that in the DTBP-0 group (3.2310 ± 0.8132 ng/L, *p* = 0.02; *n* = 6). At both E11.5d and P0.5d, no distinct differences in serum concentrations were observed between DTBP-0 and DTBP-75 groups (2.0826 ± 0.1105 vs. 2.1590 ± 0.0446 ng/L, *p* = 0.54; *n* = 5; 2.7585 ± 0.3288 vs. 2.6310 ± 0.5875 ng/L, *p* = 0.85; *n* = 6).

To investigate whether 2,4-DTBP accumulates in the embryos of offspring, this study measured the concentrations in whole embryos of offspring at E11.5d and E16.5d (Figure 5b). At E11.5d, although maternal blood 2,4-DTBP levels did not significantly increase, the 2,4-DTBP concentrations in the embryos of the DTBP-75 group (26.2338 ± 2.0290 ng/g; *n* = 5) were significantly higher than DTBP-0 group (20.6153 ± 1.3148 ng/g; *n* = 6; *p* = 0.04). At E16.5d, 2,4-DTBP further accumulated in the DTBP-75 group of embryos, reaching a concentration of 45.2662 ± 5.9877 ng/g (*n* = 6), whereas 12.8282 ± 0.6417 ng/g was detected in the DTBP-0 group (*n* = 6; *p* < 0.001).

Quantitative detection of 2,4-DTBP in major organ tissues (heart, kidney, liver, brain, and lung) of P0.5d offspring was conducted (*n* = 6; Figure 5c). In the DTBP-75 group, 2,4-DTBP concentrations were relatively high in the heart, kidney and liver, but relatively low in the brain and lung. The levels of 2,4-DTBP in the liver and brain of the DTBP-75 group (64.7153 ± 7.3854 ng/g; 38.8115 ± 2.9575 ng/g, respectively) were significantly greater than DTBP-0 group (33.0393 ± 1.7158 ng/g, *p* < 0.01; 22.0303 ± 1.3028 ng/g, *p* < 0.001, respectively). 2,4-DTBP also tended to accumulate in kidneys in DTBP-75 group (79.9823 ± 11.5795 ng/g), though the difference was not statistically significant (64.3522 ± 6.5825 ng/g in DTBP-0 group, *p* = 0.27).

### 3.4. Intrauterine Exposure to 2,4-DTBP Increased the Risk of CAKUT in Offspring

**Figure 6:**
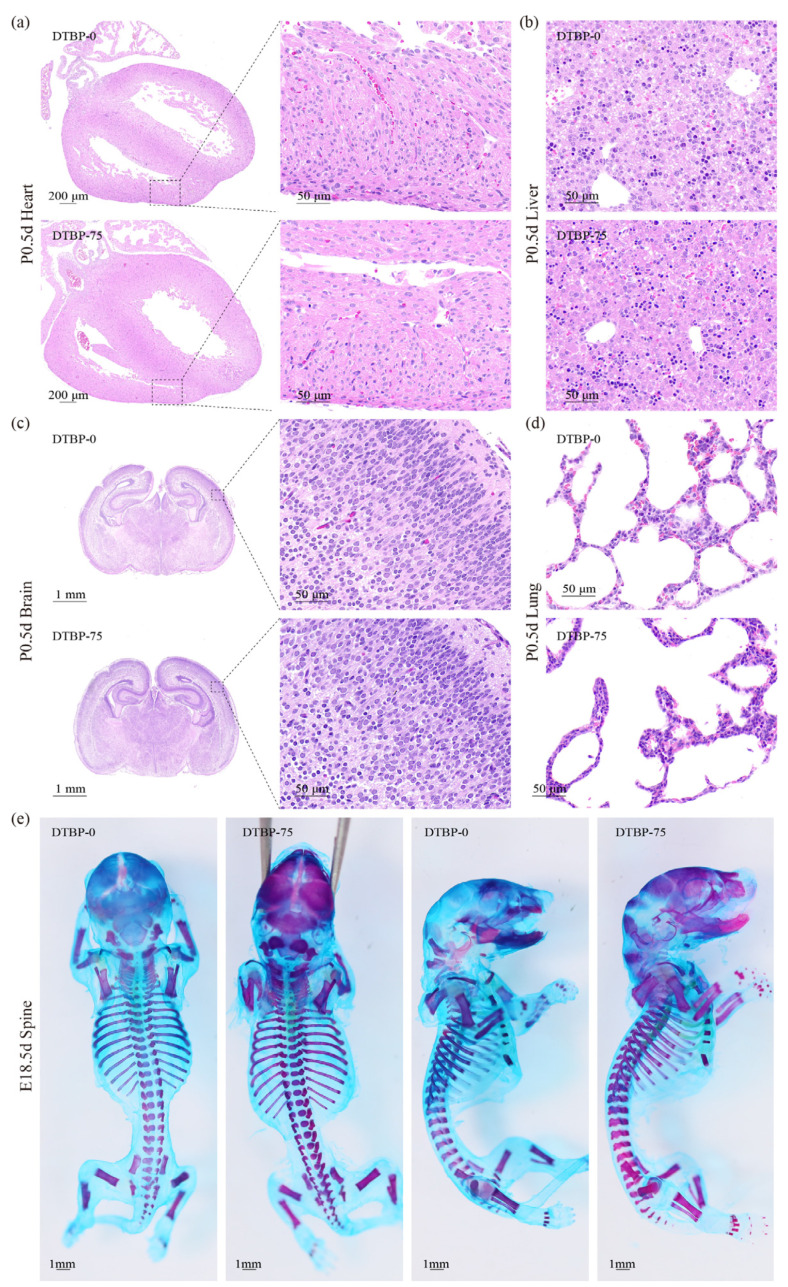
Hematoxylin–eosin-stained sections of heart (**a**), liver (**b**), brain (**c**) and lung (**d**) from P0.5d offspring. (**e**) Alcian blue and alizarin red stained skeleton of E18.5d embryo.

Morphological and pathological observations of P0.5d mice organs revealed an increased risk of CAKUT in offspring from the 2,4-DTBP exposed groups. Distinct cardiac pathologies, including hypertrophy and myocardial disarray, were observed in the DTBP-75 group (Figure 6a). Hepatic, cerebral, and pulmonary development exhibited no significant abnormalities (Figure 6b–d), nor did skeletal morphogenesis in E18.5 embryos (Figure 6e).

**Figure 7:**
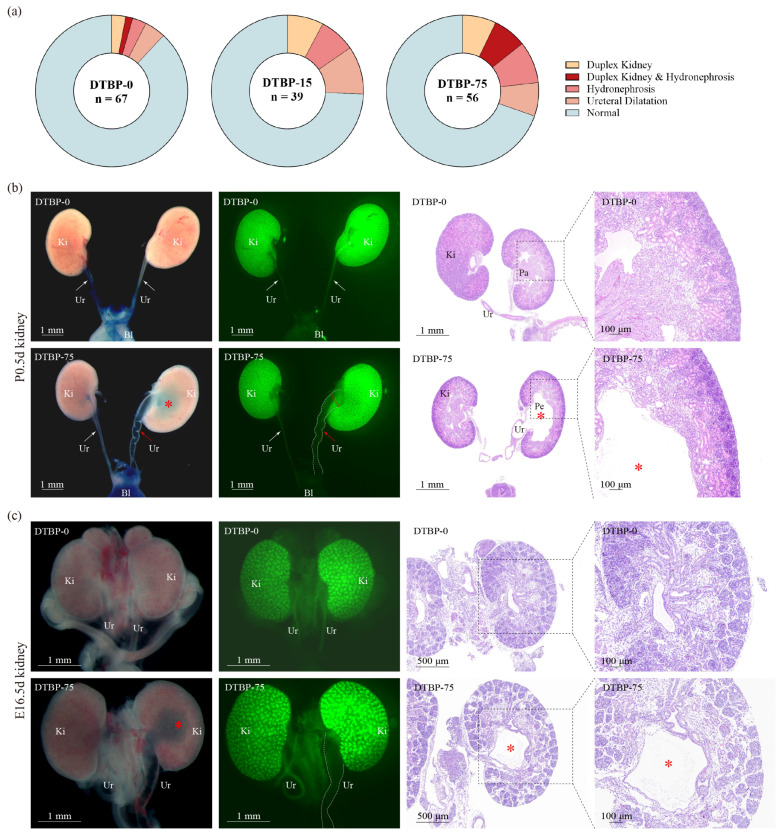
Kidney development at P0.5d and E16.5d. (**a**) Incidence and phenotypic spectrum of CAKUT in P0.5d offspring. The representative images and hematoxylin–eosin-stained sections of kidney from P0.5d (**b**) and E16.5d (**c**) offspring. Ki, Kidney; Ur, Ureter; Bl, Bladder; Pa, Papillae; Pe, Pelvis. The white dotted line indicates a dilated tortuous ureter. The red asterisk indicates a dilated pelvis. The double dotted lines highlight the measurement site for the ureteral diameter at the renal pelvic junction.

A greater proportion of CAKUT was observed in the DTBP-75 group than in the DTBP-0 group (30.36% vs. 11.94%, *p* = 0.01; Figure 7a, Table S1). The incidence of duplex kidney increased from 4.48% to 14.29%, whereas the proportion of hydronephrosis/ureteral dilation increased from 8.96% (4.48% for hydronephrosis, 4.48% for ureteral dilation) to 23.21% (16.07% for hydronephrosis, 7.14% for ureteral dilation). The DTBP-15 group also exhibited a non-significant, albeit higher, prevalence of CAKUT (25.64% vs. 11.94%, *p* = 0.11; Figure 7a), with the incidence of duplex kidney increased to 7.69%, and the frequency of hydronephrosis/ureteral dilation increased to 17.95% (7.69% for hydronephrosis, 10.26% for ureteral dilation).

To account for potential litter effects in the animal experiments, the CAKUT incidence was calculated at the litter level and analyzed using GEE clustered by litter (Supplementary Materials File S2). The CAKUT incidence in the DTBP-75 group was 33.59% (95% CI: 17.62–49.56%; N = 9), compared to 11.85% (95% CI: 2.43–21.28%; N = 9) in the DTBP-0 group (*p* = 0.02), RR = 2.53 (95% CI: 1.18–5.42). Sex-stratified analysis revealed the following patterns: in female offspring, CAKUT incidence was 21.29% versus 15.42% (RR = 2.10, 95% CI: 0.84–5.24, *p* = 0.11); in male offspring, the incidence was 42.92% versus 8.75% (RR = 3.59, 95% CI: 1.25–10.33, *p* = 0.02). For each specific phenotype (duplex kidney, hydronephrosis, and ureteral dilation) within each sex stratum, we report the unweighted mean litter proportion (DTBP-75 vs. DTBP-0), GEE-derived RR with 95% CI, raw *p*-value, and adjusted *p*-values (Bonferroni, Holm, and Benjamini–Hochberg FDR). The complete dataset is available in the Supplementary Materials. Following Bonferroni correction for multiple testing across the three phenotypes, the adjusted results showed, for hydronephrosis, RR = 3.58 (*p*_raw = 0.03, *p*_adj = 0.09); for ureteral dilation, RR = 2.55 (*p*_raw = 0.04, *p*_adj = 0.12).

Microscopic examination revealed tortuous ureters, dilated lumens, and increased transparency of the renal pelvis in the DTBP-75 group of mice (Figure 7b). H&E staining revealed the disappearance of renal papillae, widening of the renal pelvis, and thinning of the renal cortex (Figure 7b). Given that VUR is a common cause of hydronephrosis, reflux testing was performed on DTBP-75 group mice exhibiting hydronephrosis. Methylene blue dye was observed refluxing along the ureters into the kidneys, spreading throughout the renal pelvis. Even the contralateral kidney, which appeared structurally normal, also showed reflux (Figure 7b). Observations of E16.5d mouse kidneys, a stage in which fetal mice have just begun producing urine, revealed evident ureteral dilation and hydronephrosis (Figure 7c), providing additional evidence that these effects are caused by developmental abnormalities during the embryonic stage.

## 4. Discussion

Our findings demonstrate that maternal exposure to 2,4-DTBP during pregnancy is associated with an elevated risk of pregnancy loss in mice, specifically during the early gestational stage, with no further increase in this risk observed thereafter. In successfully pregnant mice, 2,4-DTBP accumulated in maternal serum and could be transmitted to offspring embryos and visceral organs, thereby increasing the risk of CAKUT in progeny. The predominant CAKUT manifestations included hydronephrosis and ureteral dilation.

Our study revealed that exposure to 2,4-DTBP reduced pregnancy rates in mice during early pregnancy. Previous study showed that exposure to 2,4-DTBP increases the mortality of zebrafish embryos [19]. A 2024 study collected serum and chorionic villus samples from women who experienced EPL and from the corresponding DTBP-0 group and reported that SPA exposure was associated with EPL [25]. The observed discordance between the significant early pregnancy loss and the absence of subsequent reductions in litter size or embryonic weight in ongoing pregnancies in our study can be explained by the stage-specific susceptibility of gestation. The early gestational period constitutes a critical window for implantation and placental development, rendering it especially vulnerable to environmental insults that may precipitate pregnancy loss [26]. Notably, the pregnancy loss in this study appeared to follow an “all-or-none” pattern; dams that did not experience EPL maintained normal litter sizes, raising the possibility that inter-individual variation in the placental transfer efficiency of 2,4-DTBP from maternal blood might underlie this phenomenon. This remains an intriguing question worthy of future investigation. As gestation progresses to the mid-stage, characterized by active organogenesis, chemical exposures are more likely to manifest as structural malformations in surviving embryos [26]. This pattern aligns with our observations of CAKUT in the absence of effects on litter size. By late gestation, when major organ formation is largely complete, external disturbances typically exert more influence on fetal growth metrics rather than on pregnancy continuation [27,28]. Our study did not detect a significant effect of 2,4-DTBP exposure on offspring body size.

To confirm the validity of our model, and to investigate whether 2,4-DTBP accumulates in maternal serum following prenatal exposure, the serum levels of 2,4-DTBP in maternal mice were measured. Liu et al. [12] quantified serum 2,4-DTBP levels in a human cohort, with concentrations ranging from below the method quantification limit (MQL) to 14.8 ng/mL. The maternal blood concentration of 2,4-DTBP in this study model fell within this range, making it relevant for real-world pollutant-related research. Our study demonstrated that 2,4-DTBP accumulated in maternal serum throughout gestation and decreased postpartum. It is generally accepted that pregnancy induces complex and widespread changes in the metabolism of females, such as lipid accumulation (with increased lipogenesis and decreased lipolysis), followed by rapid recovery postpartum [29,30]. The metabolic process of 2,4-DTBP in the body is not currently well understood, but its accumulation in maternal blood from early to mid-pregnancy and rapid decline at P0.5d may be related to the absence of additional 2,4-DTBP exposure at P0.5d or its lipophilic properties.

Previous studies have shown that 2,4-DTBP has moderate placental transfer ability [15]. Our study confirmed that 2,4-DTBP can be transmitted from maternal exposure during pregnancy to the offspring, reaching the embryonic tissues and subsequently entering internal organs. The distribution of 2,4-DTBP in offspring organs in our study is generally consistent with the findings of Björvang et al. [31], who analyzed persistent organic pollutants (POPs) in tissues from stillborn fetuses and paired maternal blood samples. Their study revealed that POPs tend to accumulate more readily in the liver and heart, followed by the lung and brain. The most frequently detected chemicals in their study were hexachlorobenzene (HCB) and dichlorodiphenyldichloroethylene (p, p′-DDE), with Log Kow values of 5.86 and 6, respectively. Given that 2,4-DTBP has a Log Kow value of 5.33, similar to these substances, it is hypothesized that its tissue distribution may also be similar. Although the kidney was not analyzed in Björvang et al.’s study, our research revealed that the degree of 2,4-DTBP accumulation in the mouse kidney was comparable to that in the liver and heart.

Although research on the metabolic pathways of 2,4-DTBP is still insufficient, insights can be drawn from its structural analog, 2,6-di-tert-butyl-hydroxytoluene (BHT). Both compounds are SPAs and studies in mice show that BHT undergoes oxidation of the tert-butyl groups and aromatic ring, resulting in significant hepatic and renal accumulation [32]. Given that similarly high concentrations of 2,4-DTBP have been found in human urine [13], it likely undergoes a comparable metabolic pathway and is excreted renally. This suggests that exposure to 2,4-DTBP may present a potential toxicological risk to the kidneys.

Adverse intrauterine environments are believed to play a critical role in the embryonic development of the kidneys [2]. Maternal exposure to bisphenol A in mice, as well as exposure to perfluorooctane sulfonate or atrazine during pregnancy in rats, has been shown to reduce the number of nephrons in offspring [33,34]. Additionally, maternal exposure to heavy metals in drinking water during pregnancy and lactation in rats can cause congestion in the renal cortex and medulla of offspring [35]. Using litter as the unit for descriptive summaries and population-averaged GEE for RR with clustering by litter, the results of this study indicated that intrauterine exposure to 2,4-DTBP is associated with the increased risk of CAKUT in offspring. The effect persists when summarized by per-litter proportions and when modeled with a log-link GEE. Sex-stratified results are directionally consistent with the overall effect; however, precision varies across strata, and some strata are limited by sample size. As for phenotypes, hydronephrosis and ureteral dilation emerged as the predominant malformations in offspring, with both demonstrating elevated risk ratios (3.58 and 2.55, respectively) prior to multiple testing correction. Following Bonferroni correction, these associations showed strong trends toward significance (*p*_adj = 0.09 and 0.12, respectively), suggesting biological effects that warrant further investigation with larger sample sizes. Regrettably, the CAKUT incidence in the DTBP-0 group in this study was 11.85%, which may be due to the developmental toxicity of DMSO. A previous study reported a hydronephrosis rate of 16.20% in offspring from mice treated with DMSO at a dose of 0.1 µL/g·day by gavage for at least 15 days prior to mating and throughout pregnancy [24].

This study has certain limitations that should be considered. The CAKUT incidence in the DTBP-0 group was 11.85%, substantially higher than the reported baseline of approximately 5% [36], implicating the use of a 1% DMSO vehicle as a primary constraint in this study. The potential confounding effect of DMSO, together with litter effects, represents a key limitation that complicates the interpretation of results and warrants careful attention in future experimental designs. To unequivocally attribute the observed renal effects to 2,4-DTBP, subsequent studies should employ alternative vehicles devoid of DMSO. Furthermore, although renal phenotypes were observed in offspring after maternal exposure to 2,4-DTBP of 75 and 15 µg/g·day during gestation, subsequent LC-MS analysis of serum and tissues was confined to the DTBP-75 group, which exhibited more pronounced effects. This dose-dependent pattern corroborates findings in zebrafish embryos, where 2,4-DTBP also induced more severe effects, such as spinal curvature and yolk sac edema, at higher concentrations [19]. The consistency of this dose–response relationship across different species strengthens the evidence for the intrinsic hazardous potential of 2,4-DTBP. It is therefore hypothesized that a dose-dependent mechanism in maternal–fetal transfer and bioaccumulation accounts for the differential phenotypic incidence. To better understand the risks at real-world exposure levels, future dose–response studies should include lower, environmentally relevant concentrations. Furthermore, it remains worthwhile to investigate whether renal tissue concentrations of 2,4-DTBP correlate with specific phenotypic outcomes. Such data are crucial for informing environmental monitoring and guiding the development of preventive measures against this emerging pollutant.

Regarding the potential mechanisms, it is noteworthy that researchers have reported that 2,4-DTBP treatment reduces TBX6 transcript levels in human-induced pluripotent stem cells [37]. Insufficient dosage of the TBX6 gene has been identified as a potential driver of CAKUT [38], highlighting the need for further investigation into the relationship between 2,4-DTBP and TBX6. Additionally, crystal structure analysis and in vitro experiments have shown that 2,4-DTBP directly binds to and activates retinoid X receptor α (RXRα) [39]. The retinoic acid (RA) signaling pathway is crucial for kidney embryogenesis, which relying on paracrine RA signaling between ureteric bud and stromal mesenchyme. RA signaling is vital for both ureteric bud formation and the development of the collecting duct system [40]. It is therefore hypothesized that 2,4-DTBP may also disrupt normal kidney development by interfering with the RA signaling pathway. The mechanisms underlying 2,4-DTBP-induced elevation of CAKUT risk require further investigation in future studies. Analyzing the expression profiles of TBX6 and genes associated with ureteric bud branching morphogenesis could help elucidate the transcriptional mechanisms underlying the observed renal defects. Subsequently, investigating RXRα antagonism or RA modulation during organogenesis would help delineate the role of RA signaling disruption in 2,4-DTBP-induced CAKUT.

## 5. Conclusions

A maternal mouse model exposed to 2,4-DTBP throughout pregnancy was established by gavage. Exposure to 2,4-DTBP during pregnancy was associated with an increased incidence of EPL, but did not significantly affect the number or size of offspring in successful pregnancies. 2,4-DTBP could accumulate in the maternal mice and be transmitted to the offspring and their internal organs, concomitant with a higher incidence of CAKUT, primarily manifested as hydronephrosis/ureteral dilation. It should be noted that these findings were observed under conditions involving a DMSO-based vehicle, the potential influence of which cannot be excluded. Furthermore, the conclusions drawn here would benefit from a more robust statistical approach in future studies to better account for litter effects and other confounding variables.

## Figures and Tables

**Table 1:** The parameters of LC.

Item	Parameters
mobile phase A	ultrapure water + 0.05 mM ammonium acetate
mobile phase B	methanol
injected sample volume	1 µL
flow rate	0.3 mL/min
column temperature	40 °C

**Table 2:** Elution Gradient of LC.

Time	Composition of Phase A	Composition of Phase B
0 min	45%	55%
1.5 min	45%	55%
7.5 min	0%	100%
13.0 min	0%	100%
13.1 min	45%	55%
17.0 min	45%	55%

**Table 3:** The parameters of electrospray ionization (ESI) source and the MRM transition for 2,4-DTBP.

Item	Parameters
gas 1	40 psi
gas 2	70 psi
curtain gas	40 psi
cad gas	9
source temperature	600 °C
polarity	negative
quantitative transition (CE ^1^, CXP ^2^)	205.1 > 189.1 (−34 V, −20 V)
confirmation transition (CE, CXP)	205.1 > 173.1 (−54 V, −15 V)

^1^ CE: collision energy, ^2^ CXP: collision cell exit potential.

## Data Availability

All processed data and STATA code used for the statistical analysis are available from the corresponding author upon reasonable request.

## Supplementary Materials

The following supporting information can be downloaded at https://www.mdpi.com/article/10.3390/toxics13110991/s1, File S1; File S2; Figure S1: Representative chromatogram. (a) Total Ion Chromatogram (TIC) of the standard compound. (b) Extracted Ion Chromatogram (EIC) of the standard compound; Figure S2: Liquid chromatography–tandem mass spectrometry (LC-MS) chromatogram of different samples; Table S1: The occurrence of CAKUT in postnatal 0.5 day (P0.5d) offspring mice.

## Abbreviations

The following abbreviations are used in this manuscript:

CAKUT	congenital anomalies of the kidney and urinary tract
EDCs	endocrine-disrupting chemicals
2,4-DTBP	2,4-Di-tert-butylphenol
RR	relative risk
SPA	synthetic phenolic antioxidant
NOAEL	No-Observed-Adverse-Effect Level
DMSO	dimethyl sulfoxide
PEG 300	polyethylene glycol 300
Tween 80	polysorbate 80
E0.5d	embryonic 0.5 day
P0.5d	postnatal 0.5 day
LC-MS	liquid chromatography–tandem mass spectrometry
MRM	multiple reaction monitoring
ESI	electrospray ionization
CE	collision energy
CXP	collision cell exit potential
H&E	hematoxylin–eosin
VUR	vesicoureteral reflux
GEE	generalized estimating equations
LMM	linear mixed-effects model
SE	standard error
CI	confidence interval
ns	non-significant
EPL	early pregnancy loss
ALT	alanine transaminase
AST	aspartate aminotransferase
ALB	albumin
BUN	blood urea nitrogen
CREA	creatinine
UA	uric acid
MQL	method quantification limit
POPs	persistent organic pollutants
HCB	hexachlorobenzene
p, p′-DDE	dichlorodiphenyldichloroethylene
BHT	2,6-di-tert-butyl-hydroxytoluene
RXRα	retinoid X receptor α
RA	retinoic acid
