# Supplementary material for: Maternal Exposure to 2,4-Di-tert-butylphenol During Pregnancy in a Mouse Model Leads to Abnormal Development of the Urinary System in Offspring
Source: Toxics. 2025 Nov 18;13(11):991. doi: 10.3390/toxics13110991 (PMC12656196; doi:10.3390/toxics13110991)
Supplement: Supplementary file 1 [file toxics-13-00991-s001.zip › File S1.pdf]

Supplementary Material S1

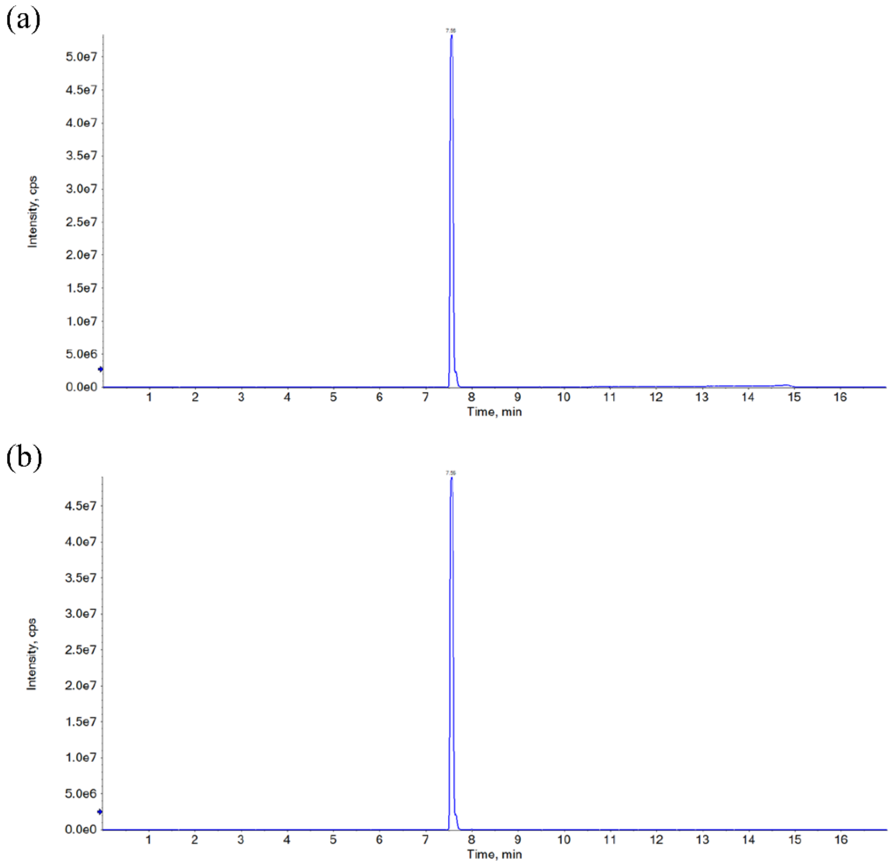

Figure S1. Representative chromatogram. (a) Total Ion Chromatogram (TIC) of the standard compound. (b) Extracted Ion Chromatogram (EIC) of the standard compound.

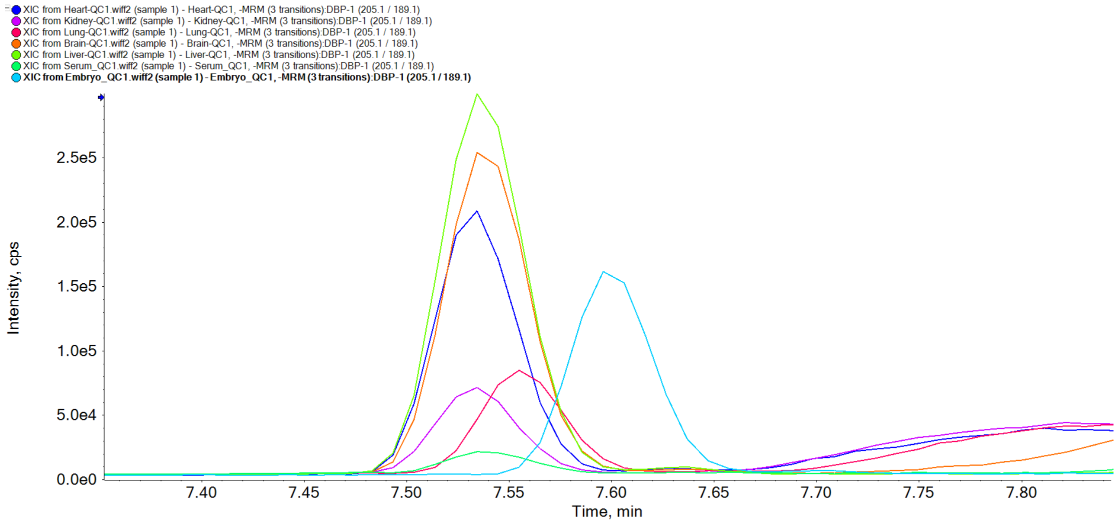

Figure S2. Liquid chromatography-tandem mass spectrometry (LC-MS) chromatogram of different samples.

Table S1. The occurrence of CAKUT in postnatal 0.5 day (P0.5d) offspring mice.

Group	Number of Each Litter	CAKUT			
		Duplex Kidney	Duplex Kidney & Hydronephrosis	Hydronephrosis	Ureteral Dilatation
DTBP-0	6	2			
	10		1		1
	6				1
	9				
	6			1	
	7				
	3				
	10			1	1
	10				
DTBP-15	7	1			
	7	1		1	
	8			2	1
	6				2
	2				1
	5				
	4	1			
DTBP-75	4			1	1
	5	2			1
	7				1
	8	1			
	9			2	
	6	1	2		
	5		1	2	
	6		1		
	6				1
